# Activation of Prefrontal Cortex in Process of Oral and Finger Shape Discrimination: fNIRS Study

**DOI:** 10.3389/fnins.2021.588593

**Published:** 2021-02-05

**Authors:** Noriyuki Narita, Kazunobu Kamiya, Sunao Iwaki, Tomohiro Ishii, Hiroshi Endo, Michiharu Shimosaka, Takeshi Uchida, Ikuo Kantake, Koh Shibutani

**Affiliations:** ^1^Research Institute of Oral Science, Nihon University School of Dentistry at Matsudo, Matsudo, Japan; ^2^Department of Removable Prosthodontics, Nihon University School of Dentistry at Matsudo, Matsudo, Japan; ^3^Mental and Physical Functions Modeling Group, Human Informatics and Interaction Research Institute, National Institute of Advanced Industrial Science and Technology, Tsukuba, Japan; ^4^Physical Fitness Technology Group, Human Informatics and Interaction Research Institute, National Institute of Advanced Industrial Science and Technology, Tsukuba, Japan; ^5^Department of Anesthesiology, Nihon University School of Dentistry at Matsudo, Matsudo, Japan; ^6^Dental Support Co., Ltd., Chiba, Japan

**Keywords:** prefrontal cortex, parallel activity, serial activity, oral, finger, shape discrimination

## Abstract

**Background:**

The differences in the brain activities of the insular and the visual association cortices have been reported between oral and manual stereognosis. However, these results were not conclusive because of the inherent differences in the task performance-related motor sequence conditions. We hypothesized that the involvement of the prefrontal cortex may be different between finger and oral shape discrimination. This study was conducted to clarify temporal changes in prefrontal activities occurring in the processes of oral and finger tactual shape discrimination using prefrontal functional near-infrared spectroscopy (fNIRS).

**Methods:**

Six healthy right-handed males [aged 30.8 ± 8.2 years (mean ± SD)] were enrolled. Measurements of prefrontal activities were performed using a 22-channel fNIRS device (ETG-100, Hitachi Medical Co., Chiba, Japan) during experimental blocks that included resting state (REST), nonsense shape discrimination (SHAM), and shape discrimination (SHAPE).

**Results:**

No significant difference was presented with regard to the number of correct answers during trials between oral and finger SHAPE discrimination. Additionally, a statistical difference for the prefrontal fNIRS activity between oral and finger shape discrimination was noted in CH 1. Finger SHAPE, as compared with SHAM, presented a temporally shifting onset and burst in the prefrontal activities from the frontopolar area (FPA) to the orbitofrontal cortex (OFC). In contrast, oral SHAPE as compared with SHAM was shown to be temporally overlapped in the onset and burst of the prefrontal activities in the dorsolateral prefrontal cortex (DLPFC)/FPA/OFC.

**Conclusion:**

The prefrontal activities temporally shifting from the FPA to the OFC during SHAPE as compared with SHAM may suggest the segregated serial prefrontal processing from the manipulation of a target image to the decision making during the process of finger shape discrimination. In contrast, the temporally overlapped prefrontal activities of the DLPFC/FPA/OFC in the oral SHAPE block may suggest the parallel procession of the repetitive involvement of generation, manipulation, and decision making in order to form a reliable representation of target objects.

## Introduction

Irving (1968) noted that stereognosis can be defined as the ability to recognize objects using only tactile (somatic) sensation and that it particularly has been applied to the finger and mouth.

The neurophysiological mechanisms for finger stereognosis have been investigated intensively, in which the participation of fronto-parietal networks was observed in the process of haptic shape recognition (Shomstein, 2012; Katsuki and Constantinidis, 2014; Sathian, 2016; Xu et al., 2020). These results suggest that a top-down bias attention may be advantageous for familiar percepts in haptic shape recognition, whereas a bottom-up bias attention may be more reliable under circumstances with unfamiliar percepts (Sathian, 2016; Xu et al., 2020) or during unskilled task performance (Lee et al., 2007; Jackson and Raymond, 2008; Seidler et al., 2012). In contrast, the neural mechanisms for oral stereognosis have not been thoroughly studied despite its importance in cognitive aging confirmed in many clinical and behavioral studies, where denture wearing in the elderly population has been shown to be effective in improving oral stereognosis (Müller et al., 1995; Jacobs et al., 1998; Ikebe et al., 2007; Meenakshi et al., 2014; Singh and Mattoo, 2014; Fukutake et al., 2019).

One of the few studies on brain activities during oral stereognosis employed functional near-infrared spectroscopy (fNIRS) to report that activities in the prefrontal cortex decreases with age (Kawagishi et al., 2014); however, it is still not clear whether the prefrontal activities during oral stereognosis differ from those during finger stereognosis. Another neuroimaging study by Fujii et al. (2011) compared differences in the brain activities between finger and oral stereognosis directly by using functional magnetic resonance imaging (fMRI), which presented the predominant activities in the insular and the visual association cortices during oral stereognosis; however, the results are not conclusive probably because the effects from the differences in the motor task sequences between the oral and finger shape discrimination tasks were not controlled adequately.

We previously clarified the cross-modal associations between oral somatosensory and visual cortex activities during oral shape discrimination where the effects of motor sequence during the task performance were compensated by the SHAM condition, under which participants mimic only the mouth movement without a target object (Kagawa et al., 2014). Taking into account the effects of motor sequences specific to each target task condition by using the SHAM condition for each task performance, detailed differences in the brain activities between finger and oral shape discrimination tasks should be revealed more precisely.

In the present study, we compare the brain responses to oral and finger shape discrimination conditions as characterized by the differences in the fNIRS signals during the target and SHAM task performances for each discrimination condition (Reed et al., 2004; Kagawa et al., 2014). Specifically, we focus on the fNIRS signals measured in the prefrontal cortices, which represent the manipulation of mental representation of the target (target image) and reference (reference image) objects (Cohen-Kdoshay and Meiran, 2009; Hering et al., 2018; Emch et al., 2019).

## Materials and Methods

### Participants

Six healthy right-handed males [aged 30.8 ± 8.2 years (mean ± SD)] were enrolled in this study. All subjects were mentally healthy, as indicated by a score lower than 7 on the Hospital Anxiety and Depression Scale (Zigmond and Snaith, 1983). The sample size for the two-way repeated measures ANOVA in this study was determined using the G^∗^Power 3 software package (noncommercial program downloaded from the University of Dusseldorf, Germany) (Faul et al., 2007), which established parameters with a significance level of 0.05, statistical power of 0.80, and effect size of 0.25 (medium effect). Each provided written informed consent for participation in this study, which was approved by the Ethics Committee of Nihon University School of Dentistry at Matsudo (EC 19-14-015-1).

### Experimental Procedures

**FIGURE 1:**
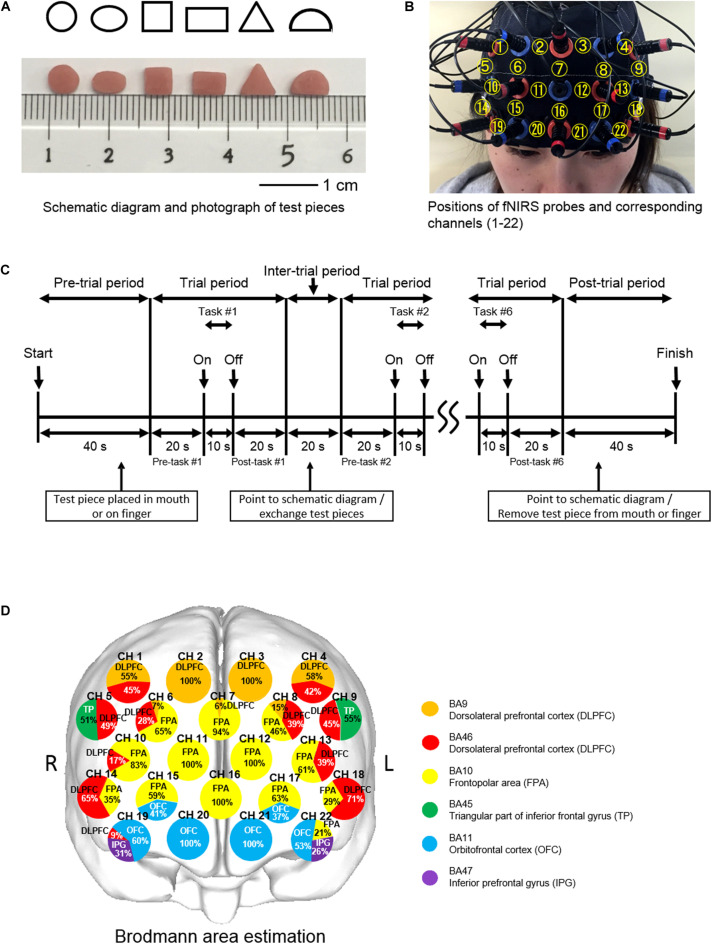
Experimental aspects of finger and oral shape discrimination using prefrontal functional near-infrared spectroscopy (fNIRS). **(A)** Schematic diagrams and photographs of six types of test pieces: circle, ellipse, square, rectangle, triangle, and semicircle. **(B)** Positioning of the prefrontal fNIRS probes and corresponding channel numbers. **(C)** Timeline of a single session composed of six trials of shape discrimination in 8 min. **(D)** Location of the prefrontal fNIRS channels. *Orange* and *red* indicate the dorsolateral prefrontal cortex (*DLPFC*); *yellow*, the frontopolar area (*FPA*); *green*, triangular part of the inferior frontal gyrus (*TP*); *blue*, the orbitofrontal cortex (*OFC*); and *purple*, the inferior prefrontal gyrus (*IFG*). Each *circle* corresponds to a channel number. *Pie chart* shows percentage in cortical areas.

Experiments were performed under the same conditions used in our previous study (Kagawa et al., 2014). Briefly, each of the six subjects was comfortably seated in a quiet room and a screen was positioned around them so as to block extra visual information from entering their field of view. The experimental blocks required six test pieces for discrimination, which were made of autopolymer resin (UNIFAST3, GC, Japan). Six test pieces were differently shaped, including a circle, ellipse, square, rectangle, triangle, and a semicircle, and each shape was tested once for each test type (oral and finger), in a random order (Kaiba et al., 2006) (Figure 1A).

Prior to the experimental blocks, the subjects received explanations regarding the procedures, including the six differently shaped test pieces and a schematic diagram, and rehearsed one block without any test pieces used (Kaiba et al., 2006).

During the pre-trial period, the investigator approached the subject from the rear so as not to enter their field of view and then instructed them to open their mouth in order to place a test piece with tweezers on the tongue or open their hands upward to place the piece on the anterior belly of the index finger. One of six test pieces was placed in the mouth preceding oral shape discrimination (O-SHAPE) or on the tip of the index finger of the right hand preceding finger shape discrimination (F-SHAPE). The subject was asked to quietly wait for verbal cues during the pre-trial and pre-task periods, after which they began to manipulate the test piece in their mouth without biting or in their hands using the index, middle, and ring fingers during the 10-s task period. After 10 s, they were given a verbal cue to stop the shape exploration and return to a quiet condition for the post-task period. The test piece was removed from the mouth or fingers during the inter-trial period of 20 s by means of tweezers. Following that, the subject was asked to point to one of the images on the schematic diagram that they thought had been explored during the task trial.

Oral shape discrimination with no test pieces (O-SHAM), or finger shape discrimination with no test pieces (F-SHAM), and Rest (REST) blocks were also conducted in this study. During the SHAM blocks, the subject was instructed to mimic their movements, with no test piece placed in the mouth or on the fingers, and then point toward the schematic diagram after the experimenter had imitated removing the test piece. During the REST block, there was no task performance and the subject was instructed to remain quiet. The O-SHAPE, F-SHAPE, O-SHAM, F-SHAM, and REST blocks were performed in random order in every subject. REST blocks were used to take account of brain activity fluctuations, with the findings obtained during that time related to brain responses specific to the SHAM and SHAPE blocks evaluated by comparing the fNIRS signals measured during each of those blocks with the signals recorded during the REST. Each block lasted for 8 min and the intervals between blocks were set at 5 min; thus, the total duration for five experimental blocks in one session was 60 min for each subject.

### Measurements of Prefrontal fNIRS Activities

Prefrontal fNIRS activities were measured according to the methods used in our previous study (Kamiya et al., 2016). Briefly, prefrontal activity was assessed during the pre-task, task, and post-task periods using a 22-channel fNIRS device (ETG-100, Hitachi Medical Co., Chiba, Japan) that utilizes two wavelengths of near-infrared light (780 and 830 nm) (Narita et al., 2009). Probes were fitted with 3 × 5 thermoplastic shells and placed on the prefrontal region, with the bottom lines of the fNIRS probes set according to FP1 and FP2, with referral to the international 10-20 system (Klem et al., 1999) (Figure 1B). fNIRS was used to determine relative changes in the concentrations of oxygenated hemoglobin ([oxy-Hb]), deoxygenated-hemoglobin ([deoxy-Hb]), and total hemoglobin. A change in [oxy-Hb] was used as an indicator of change in regional cerebral blood volume because it is more sensitive than [deoxy-Hb] as a parameter for measuring blood flow changes associated with brain activation (Hoshi et al., 2001) and has a stronger correlation with blood oxygenation level-dependent signals measured by fMRI (Strangman et al., 2002). The sampling interval was 0.1 s. During the measurements, the subjects were instructed to open their eyes and gaze at a point forward. Each trial was repeated six times, and the data obtained were averaged using the “integral mode” of the ETG-100 software package for the F-SHAPE, O-SHAPE, F-SHAM, O-SHAM, and REST blocks (Figure 1C). Also, the linear fitting algorithm (Naseer and Hong, 2015) was used for baseline correction (Ehlis et al., 2007). A moving average with a window width of 5 s was used to remove physiological noise such as cardiac artifacts (Yang et al., 2020) and the short-term motion artifacts (Suto et al., 2004; Wang et al., 2013) in the fNIRS signals.

### Location of the Prefrontal fNIRS Channel

The three-dimensional locations of each probe and the landmark position (NZ, Iz, AI, A2, Cz) on the scalp of each participant were recorded by utilizing a 3D magnetic space digitizer (3SPACE ISOTRACK2, Polhemus, United States). Furthermore, estimation of the corresponding location of each channel in the Montreal Neurological Institute (MNI) space (Collins et al., 1994; Brett et al., 2002) was obtained using a probabilistic registration method (Okamoto and Dan, 2005; Singh et al., 2005), with anatomical localization corresponding to the probe position identified using Platform for Optical Topography Analysis Tools (POTATo, Hitachi, Japan) with reference to Automated Anatomical Labeling (Tzourio-Mazoyer et al., 2002; Tsuzuki and Dan, 2014) (Figure 1D).

**FIGURE 2:**
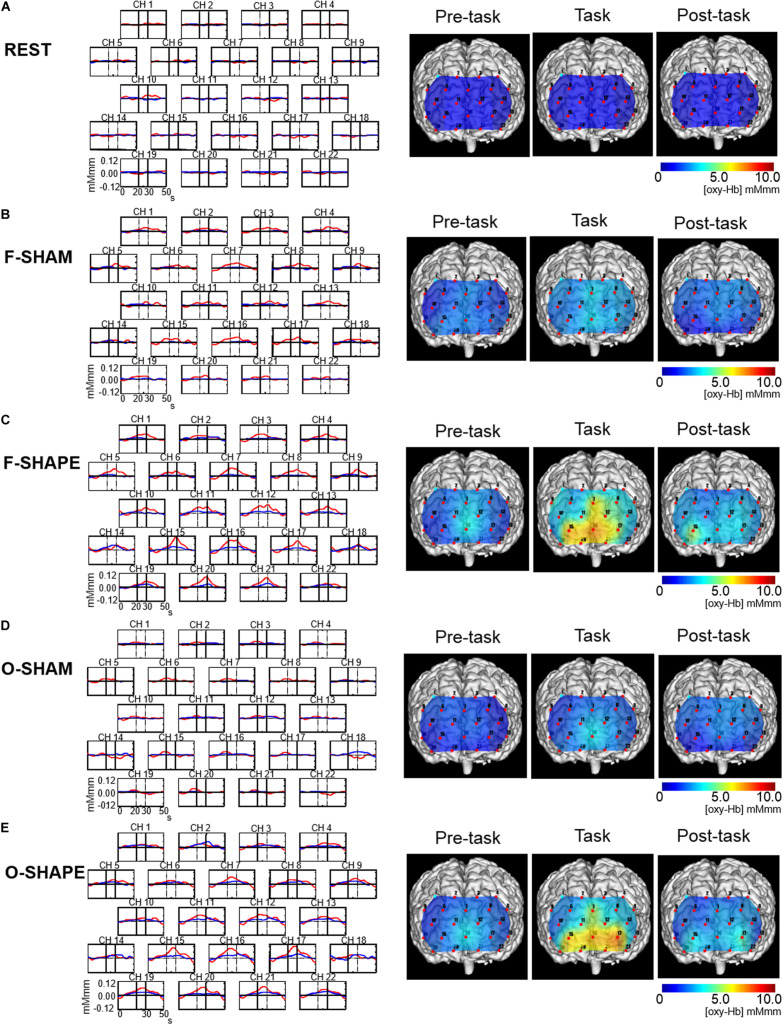
Grand average waveforms and topographical maps of the prefrontal functional near-infrared spectroscopy (fNIRS) activities. Shown on the *left* are the grand averages in the six subjects’ waveforms of oxygenated hemoglobin concentration ([oxy-Hb], *red line*) and deoxygenated hemoglobin concentration ([deoxy-Hb], *blue line*) for each of the 22 measurement channels during the REST, SHAM, and SHAPE blocks of finger and oral shape discriminations. The *x*-axis indicates time (in seconds) and the *y*-axis indicates hemodynamic changes in [oxy-Hb]. *Vertical lines* at 20 and 30 s indicate the onset and offset of the 10-s task period. Shown on the *right* are the topographical maps of the changes in [oxy-Hb] during the 10-s period preceding the task, the 10-s task period, and the 10-s period following the task period during the REST, SHAM, and SHAPE blocks of finger and oral manipulations. Changes in [oxy-Hb] during the task periods were not clearly demonstrated during the REST and SHAM blocks **(A,B,D)**, whereas it was clearly increased during the F-SHAPE and O-SHAPE **(C,E)**.

### Data and Statistical Analyses

A Wilcoxon signed-rank test was used to compare the averaged number of correct answers of the six shaped test pieces between the F-SHAPE and O-SHAPE blocks (Jacobs et al., 1997; Mantecchini et al., 1998). Two-way repeated measures ANOVA and multiple comparisons using a Bonferroni *t*-test were also applied for the time course of the averaged data for [oxy-Hb] for every 1 s during the pre-task, task, and post-task periods to compare between the SHAPE and REST, SHAM and REST, and SHAPE and SHAM blocks. The statistical software package SigmaPlot 12.5 (Systat Software Inc., AC, United States) was used for all analyses, and *p-*values less than 0.05 were considered to indicate a significant difference.

## Results

### Behavioral Performance

#### Number of Correct Answers of F-SHAPE and O-SHAPE

The number of correct answers averaged over six subjects (mean ± SD) during the F-SHAPE blocks were 4.5 ± 1.0, while those during the O-SHAPE were 4.7 ± 1.0. There was no significant difference (Wilcoxon signed-rank test: *Z* = 0.408, *p* = 0.818) shown between the numbers of correct answers by the six subjects.

### fNIRS Activities

#### Grand Averaged Waveforms and Topographical Maps of Prefrontal fNIRS Activities

The grand averaged waveforms in the six subjects for changes in [oxy-Hb] and [deoxy-Hb] during the REST, F-SHAM, F-SHAPE, O-SHAM, and O-SHAPE blocks are shown on the left side of Figure 2, while the [oxy-Hb] maps for changes in [oxy-Hb] in the pre-task, task, and post-task periods during the REST, F-SHAM, F-SHAPE, O-SHAM, and O-SHAPE blocks are presented on the right side of Figure 2. During REST, there were no apparent changes in [oxy-Hb] with regard to prefrontal fNIRS activities (Figure 2A). O-SHAM and F-SHAM showed slight increases in [oxy-Hb] during the task periods (Figures 2B,D), while there were clear increases in [oxy-Hb] during both the task and post-task periods during the O-SHAPE and F-SHAPE blocks (Figures 2C,E).

### Comparison Between F-SHAPE and O-SHAPE

In the comparison between F-SHAPE and O-SHAPE, the values for [oxy-Hb] for F-SHAPE significantly (two-way repeated measures ANOVA and Bonferroni *t*-test: *p* < 0.05, *F* = 1.755, *p* = 0.003, power of performed test = 0.852) increased as compared to O-SHAPE at fNIRS channel (CH) 1 which corresponds to the dorsolateral prefrontal cortex (DLPFC).

### Comparison Between F-SHAPE and REST

#### Temporal Differences in Prefrontal fNIRS Activities

**FIGURE 3:**
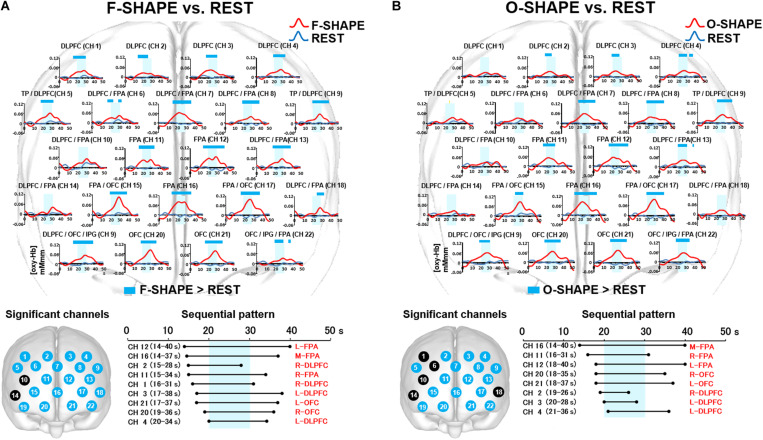
Prefrontal functional near-infrared spectroscopy (fNIRS) activities in SHAPE as compared to REST. **(A)** Temporal differences and sequential pattern of the prefrontal fNIRS activities in F-SHAPE as compared to REST. Most channels presented significant (two-way repeated measures ANOVA and Bonferroni *t*-test: *p* < 0.05) prefrontal activation in F-SHAPE as compared to REST. A sequential pattern of significant (two-way repeated measures ANOVA and Bonferroni *t*-test: *p* < 0.05) prefrontal activations also presented temporally overlapped features in the onset and duration of the dorsolateral prefrontal cortex (DLPFC)/frontopolar area (FPA)/orbitofrontal cortex (OFC) activities preceding, during, and following the task periods in F-SHAPE as compared to REST. **(B)** Temporal differences and sequential pattern of the prefrontal fNIRS activities in O-SHAPE as compared to REST. Most channels presented significant (two-way repeated measures ANOVA and Bonferroni *t*-test: *p* < 0.05) prefrontal activation in O-SHAPE as compared to REST. A sequential pattern of significant (two-way repeated measures ANOVA and Bonferroni *t*-test: *p* < 0.05) prefrontal activations also presented temporally overlapped onset and duration of the DLPFC/FPA/OFC activities preceding, during, and following the task periods in O-SHAPE as compared to REST.

Statistically significant (two-way repeated measures ANOVA and Bonferroni *t*-test: *p* < 0.05) increases in prefrontal fNIRS activities were shown by the values obtained for [oxy-Hb] in the DLPFC (CH 1–4), DLPFC/frontopolar area (FPA) (CH 6–8, 13, 18), FPA (CH 11, 12, 16), triangular part of the inferior frontal gyrus (TP)/DLPFC (CH 5, 9), FPA/OFC (CH 15, 17), OFC/inferior prefrontal gyrus (IPG)/DLPFC (CH 19), OFC (CH 20, 21), and OFC/IPG/FPA (CH 22) (Figure 3A).

#### Sequential Patterns of Prefrontal fNIRS Activities

As compared to REST, the sequential pattern of prefrontal fNIRS activities demonstrated significantly (two-way repeated measures ANOVA and Bonferroni *t*-test: *p* < 0.05) temporally overlapped features in the onset and duration of various areas of the DLPFC/FPA/OFC activities during F-SHAPE (Figure 3A).

### Comparison Between O-SHAPE and REST

#### Temporal Differences in Prefrontal fNIRS Activities

Statistically significant (two-way repeated measures ANOVA and Bonferroni *t*-test: *p* < 0.05) increases in [oxy-Hb] were noted in the DLPFC (CH 2–4), DLPFC/FPA (CH 7, 8, 13), FPA (CH 11, 12, 16), TP/DLPFC (CH 5, 9), FPA/OFC (CH 15, 17), OFC/IPG/DLPFC (CH 19), OFC (CH 20, 21), and OFC/IPG/FPA (CH 22) (Figure 3B).

#### Sequential Patterns of Prefrontal fNIRS Activities

As compared to REST, O-SHAPE significantly (two-way repeated measures ANOVA and Bonferroni *t*-test: *p* < 0.05) showed temporally overlapped features with regard to the onset and duration of prefrontal activities in the DLPFC, FPA, and OFC (Figure 3B).

### Comparison Between F-SHAM and REST

#### Temporal Differences in Prefrontal fNIRS Activities

**FIGURE 4:**
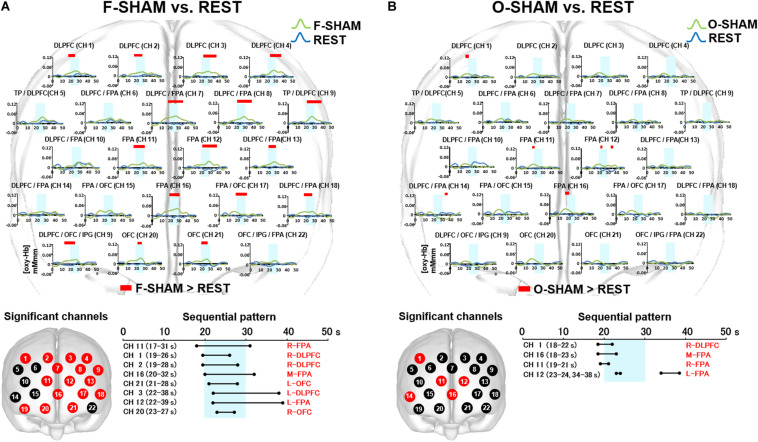
Prefrontal functional near-infrared spectroscopy (fNIRS) activities in SHAM as compared to REST. **(A)** Temporal differences and sequential pattern of the prefrontal fNIRS activities in F-SHAM as compared REST. Most channels presented significant (two-way repeated measures ANOVA and Bonferroni *t*-test: *p* < 0.05) prefrontal activation in F-SHAM as compared to REST. A sequential pattern of significant (two-way repeated measures ANOVA and Bonferroni *t*-test: *p* < 0.05) prefrontal activations presented temporally overlapped features with regard to the onset and duration of the DLPFC/FPA/OFC activities between F-SHAM and REST. **(B)** Temporal differences and sequential pattern of the prefrontal fNIRS activities in O-SHAM as compared to REST. Several channels presented significant (two-way repeated measures ANOVA and Bonferroni *t*-test: *p* < 0.05) prefrontal activations in the DLPFC and FPA in O-SHAM as compared to REST.

Statistically significant (two-way repeated measures ANOVA and Bonferroni *t*-test: *p* < 0.05) increases in [oxy-Hb] were noted in the DLPFC (CH 1–4), DLPFC/FPA (CH 7, 8, 13, 18), BA/DLPFC (CH 9), FPA (CH 11, 12, 16), FPA/OFC (CH 17), OFC/IPG/DLPFC (CH 19), and OFC (CH 20, 21) (Figure 4A).

#### Sequential Patterns of Prefrontal fNIRS Activities

As compared to REST, F-SHAM significantly (two-way repeated measures ANOVA and Bonferroni *t*-test: *p* < 0.05) showed temporally overlapped features with regard to the onset and duration of prefrontal fNIRS activities in the DLPFC, FPA, and OFC (Figure 4A).

### Comparison Between O-SHAM and REST

#### Temporal Differences in Prefrontal fNIRS Activities

Statistically significant (two-way repeated measures ANOVA and Bonferroni *t*-test: *p* < 0.05) increases in [oxy-Hb] were noted in the DLPFC (CH 1), FPA (CH 11, 12, 16), and DLPFC/FPA (CH 14) (Figure 4B). A weak association of prefrontal activities in the DLPFC and FPA was seen in O-SHAM as compared to REST.

#### Sequential Patterns in Prefrontal fNIRS Activities

As compared to REST, O-SHAM showed significant (two-way repeated measures ANOVA and Bonferroni *t*-test: *p* < 0.05) prefrontal activations in several channels in the DLPFC and FPA, and those activities diminished early during the task phase (Figure 4B).

### Comparison Between F-SHAPE and SHAM

#### Temporal Differences in Prefrontal fNIRS Activities

**FIGURE 5:**
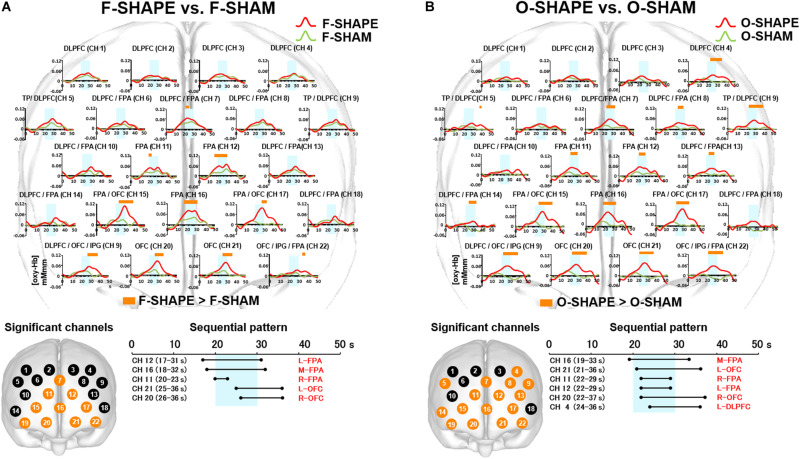
Prefrontal functional near-infrared spectroscopy (fNIRS) activities in SHAPE as compared to SHAM. **(A)** Temporal differences and sequential pattern of prefrontal fNIRS activities in F-SHAPE as compared to F-SHAM. Prefrontal activations presented significant (two-way repeated measures ANOVA and Bonferroni *t*-test: *p* < 0.05) in the frontopolar area (FPA) and orbitofrontal cortex (OFC) during F-SHAPE as compared to F-SHAM. A sequential pattern of significantly shifting features in the onset and burst was noted from the FPA to the OFC during F-SHAPE as compared to F-SHAM. **(B)** Temporal differences and sequential pattern of the prefrontal fNIRS activities in O-SHAPE as compared to O-SHAM. Most channels presented significant (two-way repeated measures ANOVA and Bonferroni *t*-test: *p* < 0.05) prefrontal activation in O-SHAPE as compared to O-SHAM. A sequential pattern of significant (two-way repeated measures ANOVA and Bonferroni *t*-test: *p* < 0.05) prefrontal activation resulted in temporally overlapped features in the onset and burst of the DLPFC/FPA/OFC activities during O-SHAPE as compared to O-SHAM.

Statistically significant (two-way repeated measures ANOVA and Bonferroni *t*-test: *p* < 0.05) increases were seen regarding the activities for [oxy-Hb] in the DLPFC/FPA (CH 7), FPA (CH 11, 12, 16), FPA/OFC (CH 15, 17), OFC/IPG/DLPFC (CH 19), and OFC (CH 20, 21) (Figure 5A).

#### Sequential Patterns of Prefrontal fNIRS Activities

As compared to SHAM, F-SHAPE findings showed that the onset and duration of prefrontal cortex activities were significantly (two-way repeated measures ANOVA and Bonferroni *t*-test: *p* < 0.05) shifted in the different prefrontal areas of the FPA and OFC. (Figure 5A). Anatomically isolated FPA activities in CH 11 (R-FPA), CH 12 (L-FPA), and CH 16 (M-FPA) preceded task onset and continued beyond the task period, whereas OFC activities in CH 20 (R-OFC) and CH 21 (L-OFC) were initiated during the middle of the task phase and lasted beyond the end of the FPA activities in the post-task period. FPA activities in the pre-task and task periods preceded the onset of OFC activities, and OFC activities in the post-task period followed the offset of FPA activities. Thus, a comparison of F-SHAPE and F-SHAM presented temporally shifting features in the onset and duration from the FPA to the OFC (Figure 5A).

### Comparison Between O-SHAPE and SHAM

#### Temporal Differences in Prefrontal fNIRS Activities

When O-SHAPE was compared with O-SHAM, statistically (two-way repeated measures ANOVA and Bonferroni *t*-test: *p* < 0.05) significant increased [oxy-Hb] were shown in the DLPFC (CH 4), TP/DLPFC (CH 5 and 9), FPA (CH 7, 11, 12, and 16), DLPFC/FPA (CH 8, 13, and 14), FPA/OFC (CH 15 and 17), OFC/IPG/DLPFC (CH 19), OFC (CH 20 and 21), and OFC/IPG/FPA (CH 22) (Figure 5B).

#### Sequential Patterns of Prefrontal fNIRS Activities

As compared to O-SHAM, O-SHAPE significantly (two-way repeated measures ANOVA and Bonferroni *t*-test: *p* < 0.05) showed temporally overlapped features with regard to the onset and duration of prefrontal activities in the DLPFC, FPA, and OFC (Figure 5B).

## Discussion

The results of the present study suggest no significant difference with regard to the number of correct answers during trials between oral and finger SHAPE discrimination as compared to REST. Additionally, a statistical difference for the prefrontal fNIRS activity between oral and finger shape discrimination was noted only in CH 1 of the DLPFC. A previous study also found no significant differences for prefrontal activities between finger and oral shape discrimination using fMRI (Fujii et al., 2011).

In the current study, we conducted randomly ordered experimental blocks of REST, nonsense SHAM, and SHAPE with six test pieces, following instructions given for the experimental procedures and a block rehearsal without any test pieces, the same as in previous studies by Reed et al. (2004) and Kagawa et al. (2014). SHAM blocks were used to compensate the differences in prefrontal activities between oral and fingers motor task sequences during shape discrimination. The differences in the prefrontal cortex activities between the SHAPE and SHAM blocks should represent the process of target image generation and manipulation in both the finger and oral shape discrimination tasks.

As results, this study presented the significant prefrontal activities in large numbers of channels between the finger SHAM and REST blocks, whereas significant prefrontal activities were only noted in a few channels between the oral SHAM and REST blocks (Figures 4A,B). Because the influences from the motor task sequences were not controlled by the use of SHAM blocks in a previous study (Fujii et al., 2011), significant differences in the prefrontal cortex activities were not observed between oral and finger shape discrimination. Further, a comparison between the finger SHAPE and SHAM showed the distinctive prefrontal activities temporally shifting from the FPA to the OFC (Figure 5A), whereas the temporally overlapped activities in the DLPFC/FPA/OFC were presented between the oral SHAPE and SHAM blocks (Figure 5B).

In consideration of the temporal hierarchical cognition process in the prefrontal cortex (Christoff and Gabrieli, 2000; Jeon, 2014; Rahnev, 2017; Gurtubay-Antolin et al., 2018), the prefrontal activities shifting from the FPA to the OFC may indicate the segregated serial prefrontal processing of tactual shape manipulation and decision making in finger shape discrimination (Daw et al., 2006; Wallis, 2007; Raposo et al., 2011; Euston et al., 2012; Laureiro-Martínez et al., 2014). In contrast, no significant difference regarding the activation of the DLPFC was found between the SHAPE and SHAM blocks in finger shape discrimination (Figures 5A,B). It has been reported that the DLPFC may be involved in monitoring and manipulating externally generated information (Christoff and Gabrieli, 2000). It is therefore assumed that the no significant difference for DLPFC activation between the finger SHAPE and SHAM blocks suggests that tactual finger shape discrimination does not require intensive DLPFC participation in generating a target image.

In contrast, as compared with the nonsense SHAM block, temporally overlapped prefrontal activities were presented in the DLPFC/FPA/OFC during the oral SHAPE block. It has been reported that the FPA is involved in the monitoring and manipulation of internal generated information, whereas the OFC is involved in the internally driven decision making (Christoff and Gabrieli, 2000). Thus, temporally overlapped prefrontal activities in the DLPFC/FPA/OFC during oral shape discrimination suggest the parallel and repetitive involvement of generation, manipulation, and decision making in order to form a reliable representation of a target object.

## Conclusion

The present results showed no significant difference with regard to the number of correct answers during trials between oral and finger SHAPE discrimination. Additionally, no statistical difference for prefrontal fNIRS activity between oral and finger shape discrimination was noted, except in CH 1. Prefrontal fNIRS activities during finger and oral shape discrimination were measured during rest, nonsense sham, and shape discrimination performed by healthy subjects. The findings indicated shifting prefrontal activities from the FPA to the OFC during finger SHAPE as compared with SHAM blocks, which suggests segregated serial processing from the manipulation of a target image to the decision making during finger shape discrimination. In contrast, the overlapped prefrontal activities in the DLPFC/FPA/OFC during oral SHAPE blocks may suggest the paralleled prefrontal processing of the repetitive involvement of generation, manipulation, and decision making in order to form a reliable representation of target objects. This study presented the characterized prefrontal activities during tactual oral and finger discrimination task performance in healthy subjects.

## Data Availability Statement

The raw data supporting the conclusions of this article will be made available by the authors, without undue reservation.

## Ethics Statement

The studies involving human participants were reviewed and approved by Ethics Committee of Nihon University School of Dentistry at Matsudo (EC 19-14-015-1). The patients/participants provided their written informed consent to participate in this study. Written informed consent was obtained from the individual(s) for the publication of any potentially identifiable images or data included in this article.

## Author Contributions

NN designed the study and wrote the manuscript. KK collected experimental data. KK and TI analyzed the obtained data. SI, HE, MS, TU, IK, and KS contributed to the interpretation of the significance of the obtained data. All authors contributed to the article and approved the submitted version.

## Conflict of Interest

TU and IK are employed by Dental Support Co., Ltd. The remaining authors declare that the research was conducted in the absence of any commercial or financial relationships that could be construed as a potential conflict of interest.
